# Th17 Cells in Protection from Tumor or Promotion of Tumor Progression

**DOI:** 10.4172/2155-9899.1000431

**Published:** 2016-06-20

**Authors:** M. Rita I. Young

**Affiliations:** 1 Research Service, Ralph H. Johnson VA Medical Center, Charleston, SC 29401, USA; 2 Department of Otolaryngology – Head and Neck Surgery, Medical University of South Carolina, Charleston, SC 29425, USA

**Keywords:** Cancer, IL-17, Th17, Inflammation, Premalignant, T-helper, Treg, Tumor

## Abstract

The roles of inflammation and inflammatory cells such as Th17 cells in the development and progression of cancer have been extensively studied. However, the results have been varied, with conflicting conclusions. Most studies have focused on changes in inflammatory phenotypes once cancers have developed and disease is progressing. Far fewer studies have looked at the immune phenotypic changes that occur during progression of premalignant lesions to cancer. The impact of inflammation and, in particular, Th17 cells on tumor biology is summarized in this review, with a focus on the differences in the outcomes of studies. Possible explanations for the contradictory conclusions are also suggested.

## Inflammation and Tumor Progression

The role of inflammation in promoting or protecting from tumor progression has been under investigation for an extensive period of time, but remains unsettled. There is literature implicating inflammation as being pro-tumorigenic in part through the induction of genetic instability [1,2]. Studies of premalignant oral lesions in patients and in a murine oral model of premalignant oral lesions have shown an increased inflammatory state that is characterized by increases in levels of inflammatory cytokines such as IL-6, TNF-α and IL-17 [3–6]. However, these studies also showed that the inflammatory state subsides as premalignant lesions progress to cancer and is replaced by inhibitory mediators. In a different carcinogen-induced mouse model for cancer development, multiple inflammatory cytokines, including IL-17, were increased during the premalignant lesion stage, but then continued to further increase once cancer developed [7]. Studies to dissect the processes associated with the linkages between inflammation and colon cancer have shown that enteropathogenic bacteria trigger pathways involving CCL2 and PGE_2_ that facilitates the recruitment and proliferation of Th17 cells in the intestine [8]. In a separate study, production of PGE_2_ by breast tumor cells was shown to stimulate dendritic cell production of IL-23 which, in turn, triggered Th17 cell expansion in the tumor microenvironment [9]. Contributing to the complexity of inflammation in cancer progression is the heterogeneity of inflammatory cells and the mediators that they can release [3,10,11]. Immune plasticity, and in particular that of CD4^+^ cells, further complicates conclusive determinations of the role of inflammation in tumor progression [4,12,13]. This is to a large part due to the plasticity being driven by the cytokine milieu, creating a highly interactive scenario whereby mediators from immune cells, premalignant lesion or tumor cells, and the surrounding stroma all having an influence on the immune infiltrate and its pro- or anti-tumorigenic effects [4,13–15].

## Are Th17 cells pro-tumorigenic or anti-tumorigenic (summary in Figure 1)?

Th17 cells are among the CD4^+^ cell populations that are inflammatory. However, they also can express Th1-type cytokines, or they can be driven toward becoming inhibitory Treg cells [4,16,17]. Results of studies to elucidate if Th17 cells promote or protect from tumor progression are particularly conflicting, with discrepancies between multiple studies. Most studies have focused on the role of Th17 in established cancer [6,15]. For examples, an increase in more highly activated Th17 cells has been associated with improved prognosis of patients with oropharyngeal squamous carcinoma [18]. Patients with benign salivary gland tumors had higher levels of Th17 cells compared to the levels in patients with malignant salivary gland tumors [19]. Studies with melanoma patients showed that those with higher levels of Th17 cells had better survival following vaccination with surviving-derived peptides [20]. Th17 cell presence in early stage ovarian cancer and in malignant pleural effusions was associated with improved prognosis as the Th17 cells could promote a Th1 cytokine environment able to recruit effector T-cells [21,22]. Consistent with this capacity to promote a Th1 environment are results of a study that looked at inter-cellular interactions that could be induced by Th17 cells [23]. This study showed that Th17 cells could induce dendritic cells that produce IL-12/IL-23 which, in turn, can polarize naïve and Th17 cells to a Th1 phenotype. In addition to their immune modulating roles, Th17 cells have also been shown to have direct anti-proliferative and apoptosis-inducing effects toward head and neck cancer [24].

Several studies have used approaches to alter levels of Th17 cells so as to determine their role in tumor growth. For example, overexpression of IL-6 in a mouse pancreatic cancer model enhanced induction of Th17 cells [25]. Associated with this increase in IL-17 cells was reduced development of cancer and improved mouse survival. A different approach using a vaccine composed of heat-shock protein 65 mixed with GL261 glioma lysate showed upregulation of IL-17 peripherally and an increase in brain-infiltrating Th17 cells [26]. Because this increase in Th17 cells was associated with prolonged survival of glioma-bearing mice, it was suggested that the Th17 cells contributed to the beneficial effects of the vaccine. A study involving several different cancer mouse models showed that treatment with mice with Toll-like receptor ligands increased the accumulation of T-cells that secreted multiple cytokines, including IL-17, delayed tumor growth and enhanced mouse survival [27]. However, this protection was absent in mice that were deficient in IL-17, further supporting the anti-tumor role of IL-17-producing cells.

Contrasting with studies suggesting a beneficial anti-cancer effect of Th17 cells, there is ample literature suggesting the opposite. Demonstration of higher levels of Th17 cells in more advanced stage hypopharyngeal carcinoma led to the suggestion that Th17 cells contribute to cancer development and metastasis [28]. In separate studies, high levels of Th17 cells were associated with poorer survival outcome in colorectal cancer and in breast cancer [29,30]. This poor clinical outcome in breast cancers with Th17 cell accumulation was shown to be associated with the inhibition of CD4^+^ cell and CD8^+^ cell activation, thus limiting anti-tumor immune reactivity [31]. While Th17 cells are typically considered to be inflammatory cells, studies with rheumatoid arthritis patients demonstrated a Th17 subset of Treg that not only produced IL-17 but also exhibiting immune inhibitory activity [32]. Whether or not such cells are also present in cancer patients has not been examined but, if present, they could contribute to tumor-induced immune inhibition and, in turn, facilitate tumor progression. There are also non-immunological mechanisms by which Th17 cells can be pro-tumorigenic. For example, by their production of IL-17, Th17 cells can enhance angiogenesis and impair the endothelial barrier, allowing for an increased tumor development and metastasis [33,34]. A separate study showed that the stimulation of angiogenesis by IL-17 was due to its stimulation of tumor cell production of VEGF [35].

## Possible explanations for contradictory conclusions

Some of the apparent contradictory roles of Th17 cells in cancer progression may be a result of how these cells are recognized, which is typically by the expression of IL-17 or by measurement of IL-17 levels as a surrogate for Th17 cells. However, a study with squamous cervical cancer showed that the main tumor-associated producers of IL-17 were not Th17 cells, but instead were neutrophils. The level of these IL-17-producing neutrophils was associated with poor disease survival while elevated levels of CD4^+^ cells expressing IL-17 were associated with improved survival [36]. A separate study with breast cancer also showed neutrophil presence to promote tumor progression, but instead showed T-cell production of IL-17 to be responsible for recruitment of these tumor-promoting neutrophils [29]. Here too, there are contradictory study conclusions. In a study with a mouse tumor model, neutrophils were shown to be protective against tumor progression by limiting the inhibitory effects of IL-17 on CD8+ T-cell responses to tumor [37]. The inter-connections between IL-17, T-cells, and neutrophils have only recently become a topic of study and much more clarity is needed before concluding how to best manipulate these interactions for therapeutic purposes.

It is possible that the apparent contradiction between protumorigenic and anti-tumorigenic effects of Th17 cells are not contradictions at all, but instead a reflection of multiple concurrent effects mediated by Th17 cells. Through their interaction with tumor and the immune infiltrate, Th17 cells can concurrently be protumorigenic and anti-tumorigenic as they can stimulate protumorigenic mediators from tumor and the tumor-associated stroma, while also recruiting anti-tumorigenic neutrophils and cytolytic Tcells [15]. Reported shifts from an inflammatory Th17 phenotype to a regulatory phenotype complicate the question of the role of Th17 cells in cancer progression. For example, increased levels of inflammatory Th17 cells have been demonstrated in gastric disease and ovarian cancer [21,38,39]. However, some of these studies show a decline in Th17 cells with a phenotypic shift to Treg cells in advanced disease, while others have shown persistence of Th17 cells. Similarly, levels of Th17-related cytokines were higher in patients with stage I to IIIA non-small cell lung cancer than in patients with more advances stage IIIB to IV cancer [40]. The progression of pancreatic cancer from an early to an advanced stage has also been shown to be associated with a shift to reduced Th17 and increased Treg cell levels [41]. What is of interest is how shifts in the frequency of Th17 cells during the course of progressive tumor growth and metastasis are interpreted. Some studies have suggested that the decline in Th17 cells is a form of tumor evasion and strategies to sustain Th17 cell levels could be tumor treatment approaches [6,21]. Other studies interpret the early presence of Th17 cells to contribute to the progressive growth and metastasis of cancer [38,39,42].

## Premalignant lesion progression to cancer

Few studies have looked at the impact of Th17 cells in the progression of premalignant lesions to cancer. A study of the impact of deficiencies in TGFβ signaling showed spontaneous development of polyps, a decline in Treg cells, and an accumulation of Th17 cells, leading to the conclusion that Th17 cells were involved in the development of the premalignant lesions [43]. Increased levels of Th17 cells and IL-17 have been demonstrated in human premalignant lesion tissue and in premalignant tongue tissue of carcinogen-treated mice, but these levels declined and were replaced with an immune inhibitory cell phenotype once oral cancers developed [3,4,14]. Such studies raise the question of whether the Th17 cells and their cytokines promoted progression from the premalignant lesion state to cancer, or whether the shift in cell phenotypes represents an attempt to limit progression to cancer, which ultimately fails. Also, it is difficult to decipher which came first, the progression from premalignant lesions to cancer or the immune shift away from a Th17 phenotype. However, *in vitro* studies have shown that the premalignant lesion cells and the premalignant lesion milieu are capable of stimulating the induction of Th17-associated cytokines, while this stimulatory capacity is lost once the lesions have progressed to cancer [4,14]. These studies also showed that soluble mediators from tumor tissue as well as primary tumor cell cultures contain high levels of TGF-β and redirect the immune phenotype toward Treg [4].

## Causality versus correlation between the Th17 phenotype and cancer development

While studies have assessed correlations between Th17 cells and cancer development, attempts to manipulate Th17 cell levels to test in a causal manner if they protect against cancer or promote cancer development are rare. One such study showed that injection of mice with IL-17, the hallmark cytokines of Th17 cells, increased tumor growth and metastasis [44]. A complementary study in a mouse mammary tumor model showed reduced development of tumor from a tumor cell inoculum in mice treated with IL-17 neutralizing antibody [29]. An alternate approach to pin down the pro- versus anti-tumorigenic impact of Th17 cells was to alter the cytokine balance so as to sustain the increased levels of Th17 cells seen in the premalignant oral lesion stage. This was accomplished with treatment with IL-23 plus a TGF-β type 1 receptor inhibitor [6]. Treatment of mice bearing carcinogen-induced premalignant oral lesions with this combination not only sustained Th17 cells, but to also increase levels of other cytokines, and slowed progression of premalignant oral lesions to cancer.

## Concluding Remarks

While studies on the immunological impact of Th17 in various environments have been increasing in scope, many of the studies are correlative associations of Th17 cell presence or IL-17 levels in the cancer setting, with far fewer studies being designed to demonstrate in a causal manner their contribution to the condition, including to cancer development and progression. Thus, there remain unanswered questions as to whether Th17 cells are beneficial or detrimental in the course of development and progression to cancer. Clearly, the answers will not be simple, but will depend on the state of tumor development and will require studies designed to demonstrate causality of associations between Th17 cell presence and tumor development stage. It will be important to note that any conclusions on the impact of Th17 cells in tumor progression will still be a snapshot in time due to the plasticity of these cells to range from inflammatory to immune suppressive phenotypes.

## Figures and Tables

**Figure 1:**
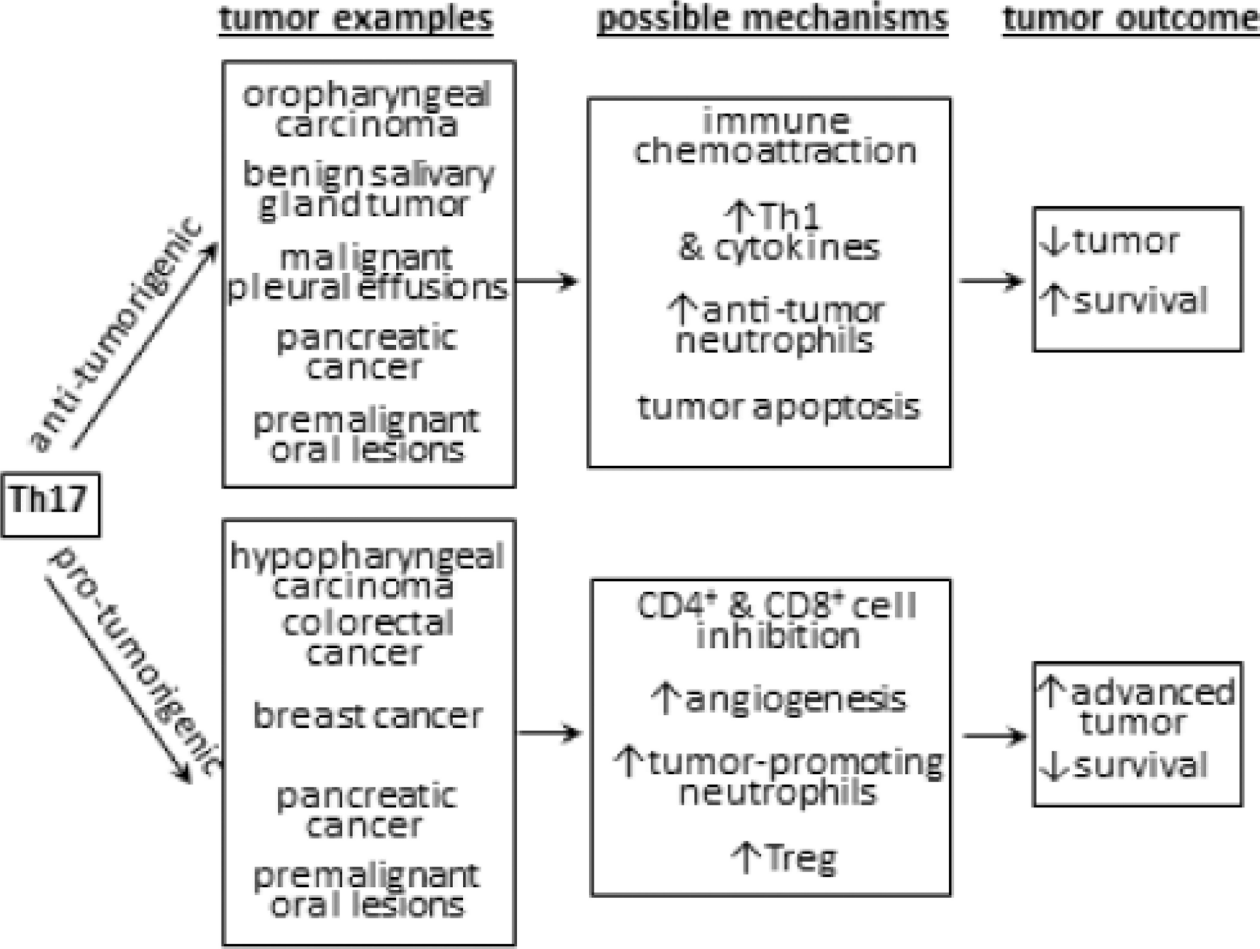
Overview of pro-tumorigenic and anti-tumorigenic activities of Th17 cells.

## References

[1] Debelec-Butuner B, Ertunc N, Korkmaz KS (2015). Inflammation contributes to NKX3.1 loss and augments DNA damage but does not alter the DNA damage response via increased SIRT1 expression. J Inflamm (Lond).

[2] Yamamoto ML, Maier I, Dang AT, Berry D, Liu J (2013). Intestinal bacteria modify lymphoma incidence and latency by affecting systemic inflammatory state, oxidative stress, and leukocyte genotoxicity. Cancer Res.

[3] De Costa AM, Schuyler CA, Walker DD, Young MR (2011). Characterization of the evolution of immune phenotype during the development and progression of squamous cell carcinoma of the head and neck. Cancer Immunol Immunother.

[4] Woodford D, Johnson SD, De Costa AM, Young MR (2014). An Inflammatory Cytokine Milieu is Prominent in Premalignant Oral Lesions, but Subsides when Lesions Progress to Squamous Cell Carcinoma. J Clin Cell Immunol.

[5] Young MR, Levingston C, Johnson SD (2015). Cytokine and Adipokine Levels in Patients with Premalignant Oral Lesions or in Patients with Oral Cancer Who Did or Did Not Receive 1α,25-Dihydroxyvitamin D3 Treatment upon Cancer Diagnosis. Cancers (Basel).

[6] Young MR, Levingston CA, Johnson SD (2016). Treatment to sustain a Th17-type phenotype to prevent skewing toward Treg and to limit premalignant lesion progression to cancer. Int J Cancer.

[7] Gasparoto TH, de Oliveira CE, de Freitas LT, Pinheiro CR, Ramos RN (2012). Inflammatory events during murine squamous cell carcinoma development. J Inflamm (Lond).

[8] Deng Z, Mu J, Tseng M, Wattenberg B, Zhuang X (2015). Enterobacteria-secreted particles induce production of exosome-like S1P-containing particles by intestinal epithelium to drive Th17-mediated tumorigenesis. Nat Commun.

[9] Qian X, Gu L, Ning H, Zhang Y, Hsueh EC (2013). Increased Th17 cells in the tumor microenvironment is mediated by IL-23 via tumor-secreted prostaglandin E2. J Immunol.

[10] Miura K, Ishioka M, Minami S, Horie Y, Ohshima S (2016). Toll-like Receptor 4 on Macrophage Promotes the Development of Steatohepatitis-related Hepatocellular Carcinoma in Mice. J Biol Chem.

[11] Orciani M, Sorgentoni G, Torresetti M, Di Primio R, Di Benedetto G (2016). MSCs and inflammation: new insights into the potential association between ALCL and breast implants. Breast Cancer Res Treat.

[12] Gagliani N, Amezcua Vesely MC, Iseppon A, Brockmann L, Xu H (2015). Th17 cells transdifferentiate into regulatory T cells during resolution of inflammation. Nature.

[13] Yu CI, Becker C, Metang P, Marches F, Wang Y (2014). Human CD141+ dendritic cells induce CD4+ T cells to produce type 2 cytokines. J Immunol.

[14] Johnson SD, De Costa AM, Young MR (2014). Effect of the premalignant and tumor microenvironment on immune cell cytokine production in head and neck cancer. Cancers (Basel).

[15] Amicarella F, Muraro MG, Hirt C, Cremonesi E, Padovan E (2015). Dual role of tumour-infiltrating T helper 17 cells in human colorectal cancer. Gut.

[16] Jimeno R, Leceta J, Garin M, Ortiz AM, Mellado M (2015). Th17 polarization of memory Th cells in early arthritis: the vasoactive intestinal peptide effect. J Leukoc Biol.

[17] Hjortsø MD, Larsen SK, Kongsted P, Met Ö, Frøsig TM (2015). Tryptophan 2,3-dioxygenase (TDO)-reactive T cells differ in their functional characteristics in health and cancer. Oncoimmunology.

[18] Punt S, Dronkers EA, Welters MJ, Goedemans R, Koljenovic S (2016). A beneficial tumor microenvironment in oropharyngeal squamous cell carcinoma is characterized by a high T cell and low IL-17 cell frequency. Cancer Immunol Immunother.

[19] Haghshenas MR, Khademi B, Faghih Z, Ghaderi A, Erfani N (2015). Immune regulatory cells and IL17-producing lymphocytes in patients with benign and malignant salivary gland tumors. Immunol Lett.

[20] Køllgaard T, Ugurel-Becker S, Idorn M, Andersen MH, Becker JC (2015). Pre-Vaccination Frequencies of Th17 Cells Correlate with Vaccine-Induced T-Cell Responses to Survivin-Derived Peptide Epitopes. PLoS One.

[21] Kryczek I, Banerjee M, Cheng P, Vatan L, Szeliga W (2009). Phenotype, distribution, generation, and functional and clinical relevance of Th17 cells in the human tumor environments. Blood.

[22] Ye ZJ, Zhou Q, Gu YY, Qin SM, Ma WL (2010). Generation and differentiation of IL-17-producing CD4+ T cells in malignant pleural effusion. J Immunol.

[23] Davidson MG, Alonso MN, Yuan R, Axtell RC, Kenkel JA (2013). Th17 cells induce Th1-polarizing monocyte-derived dendritic cells. J Immunol.

[24] Kesselring R, Thiel A, Pries R, Trenkle T, Wollenberg B (2010). Human Th17 cells can be induced through head and neck cancer and have a functional impact on HNSCC development. Br J Cancer.

[25] Gnerlich JL, Mitchem JB, Weir JS, Sankpal NV, Kashiwagi H (2010). Induction of Th17 cells in the tumor microenvironment improves survival in a murine model of pancreatic cancer. J Immunol.

[26] Yan Y, Fang M, Xuan W, Wu X, Meng X (2015). The therapeutic potency of HSP65-GTL in GL261 glioma-bearing mice. J Immunother.

[27] Marshall NA, Galvin KC, Corcoran AM, Boon L, Higgs R (2012). Immunotherapy with PI3K inhibitor and toll-like receptor agonist induces IFN-γ+IL-17+ polyfunctional T cells that mediate rejection of murine tumors. Cancer Res.

[28] Chen X, Wang J, Wang R, Su Q, Luan J (2016). Th1-, Th2-, and Th17-associated cytokine expression in hypopharyngeal carcinoma and clinical significance. Eur Arch Otorhinolaryngol.

[29] Benevides L, da Fonseca DM, Donate PB, Tiezzi DG, De Carvalho DD (2015). IL17 promotes mammary tumor progression by changing the behavior of tumor cells and eliciting tumorigenic neutrophils recruitment. Cancer Res.

[30] Yoshida N, Kinugasa T, Miyoshi H, Sato K, Yuge K (2016). A high RORγT/CD3 ratio is a strong prognostic factor for postoperative survival in advanced colorectal cancer: analysis of helper T cell lymphocytes (Th, Th2, Th17 and regulatory T cells). Ann Surg Oncol.

[31] Thibaudin M, Chaix M, Boidot R, Vegran F, Derangere V (2016). Human ectonucleotidase-expressing CD25 Th17 cells accumulate in breast cancer tumors and exert immunosuppressive functions. Oncoimmunology.

[32] Wang T, Sun X, Zhao J, Zhang J, Zhu H (2015). Regulatory T cells in rheumatoid arthritis showed increased plasticity toward Th17 but retained suppressive function in peripheral blood. Ann Rheum Dis.

[33] Kulig P, Burkhard S, Mikita-Geoffroy J, Croxford AL, Hovelmeyer N (2016). IL17A-mediated endothelial breach promotes metastasis formation. Cancer Immunol Res.

[34] Du JW, Xu KY, Fang LY, Qi XL (2012). Interleukin-17, produced by lymphocytes, promotes tumor growth and angiogenesis in a mouse model of breast cancer. Mol Med Rep.

[35] Liu J, Duan Y, Cheng X, Chen X, Xie W (2011). IL-17 is associated with poor prognosis and promotes angiogenesis via stimulating VEGF production of cancer cells in colorectal carcinoma. Biochem Biophys Res Commun.

[36] Punt S, Fleuren GJ, Kritikou E, Lubberts E, Trimbos JB (2015). Angels and demons: Th17 cells represent a beneficial response, while neutrophil IL-17 is associated with poor prognosis in squamous cervical cancer. Oncoimmunology.

[37] Liu Y, O'Leary CE, Wang LS, Bhatti TR, Dai N (2016). CD11b+Ly6G+ cells inhibit tumor growth by suppressing IL-17 production at early stages of tumorigenesis. Oncoimmunology.

[38] Maruyama T, Kono K, Mizukami Y, Kawaguchi Y, Mimura K (2010). Distribution of Th17 cells and FoxP3+ regulatory T cells in tumor-infiltrating lymphocytes, tumor-draining lymph nodes and peripheral blood lymphocytes in patients with gastric cancer. Cancer Sci.

[39] Zhong F, Cui D, Tao H, Du H, Xing C (2015). IL-17A-producing T cells and associated cytokines are involved in the progression of gastric cancer. Oncol Rep.

[40] Liao C, Yu ZB, Meng G, Wang L, Liu QY (2015). Association between Th17-related cytokines and risk of non-small cell lung cancer among patients with or without chronic obstructive pulmonary disease. Cancer.

[41] Wang X, Wang L, Mo Q, Dong Y, Wang G (2015). Changes of Th17/Treg cell and related cytokines in pancreatic cancer patients. Int J Clin Exp Pathol.

[42] Zhang W, Tian X, Mumtahana F, Jiao J, Zhang T (2015). The existence of Th22, pure Th17 and Th1 cells in CIN and cervical cancer along with their frequency variation in different stages of cervical cancer. BMC Cancer.

[43] Hahn JN, Falck VG, Jirik FR (2011). Smad4 deficiency in T cells leads to the Th17-associated development of premalignant gastroduodenal lesions in mice. J Clin Invest.

[44] Wei L, Wang H, Yang F, Ding Q, Zhao J (2016). Interleukin-17 potently increases non-small cell lung cancer growth. Mol Med Rep.

